# Effect of Marinating on Selected Quality Characteristics of Pork Tenderloin Cooked by Sous Vide Method

**DOI:** 10.3390/foods14111958

**Published:** 2025-05-30

**Authors:** Marian Gil, Mariusz Rudy, Paulina Duma-Kocan, Renata Stanisławczyk, Aleksandra Wolińska, Anna Krajewska, Dariusz Dziki

**Affiliations:** 1 Department of Agricultural Processing and Commodity Science, Institute of Food Technology and Nutrition, Faculty of Technology and Life Sciences, University of Rzeszów, Zelwerowicza 4, 35-601 Rzeszow, Poland; mgil@ur.edu.pl (M.G.); pduma@ur.edu.pl (P.D.-K.); rstanislawczyk@ur.edu.pl (R.S.); aw123915@stud.ur.edu.pl (A.W.); 2 Department of Thermal Technology and Food Process Engineering, University of Life Sciences in Lublin, 31 Głeboka Street, 20-612 Lublin, Poland; anna.krajewska@up.lublin.pl

**Keywords:** marinating, sous vide, meat quality, pork

## Abstract

The aim of this study was to evaluate the effect of marinade type and marinating time on the physicochemical and sensory properties of pork tenderloin cooked using low-temperature, vacuum-sealed cooking. The study included marinades based on pineapple juice, red wine, kefir and a mixture of dried herbs. The assessment of the effect of marinades was based on the analysis of the color, texture, chemical composition and organoleptic properties of the meat after cooking using the sous vide method. In the experimental part, instrumental determinations of color and texture, analysis of the chemical composition and sensory assessment of the meat were carried out. Marinating for 12 h in red wine and dry marinating causes darkening of the meat. The hardness of meat marinated after 2 h increased compared to the control group; similar relationships were observed for gumminess and chewiness. However, after 12 h of marinating, the hardness of cycle 1 and hardness of cycle 2, as well as chewiness and gumminess, were significantly reduced below the level of the characteristics for the control group, except for the meat marinated in wine. Meat marinated in red wine and using the dry method received higher scores, while longer marinating resulted in more favorable scores.

## 1. Introduction

In recent years, people’s health awareness has increased, which affects their preferences for high-quality food. People demand meat and meat products that have natural ingredients that ensure the health and quality of food. In addition, more and more people prefer that preparing meals at home is easy and quick. To improve the taste and texture of meat products, marinades are often used before cooking them [1,2,3]. Marinating is a preservation method that has been used in the past. It consists in adding liquids saturated with flavors, spices and functional additives to meat products [4,5]. Marinades are flavor-infusing liquids composed of diverse elements like acidic agents (e.g., vinegar, citrus juices, or fermented dairy), oils, salts, sugars, herbs, spices, aromatic compounds, and a variety of functional additives such as phosphates [6,7]. The addition of organic acids such as citric acid, acetic acid and tartaric acid lowers the pH of meat by increasing the rate of natural proteolysis. It thus accelerates the maturation of meat by shortening the time needed for its tenderization. This effect results from the disruption of tissue integrity as the meat absorbs moisture and expands, the increase in proteolysis caused by cathepsins, and the increase in the conversion of collagen to gelatin at low pH during cooking [8,9]. There are three basic methods commonly used for marinating meat: immersion, injection, and tumbling [4,10]. The simplest and most commonly used method is immersion, which can be used by meat companies of any size (small, medium, or large) and even at home, as it requires special equipment [4]. Pieces of meat are immersed in an aqueous solution with various ingredients, such as salt, organic acids, etc., and the ingredients gradually permeate by osmosis [11].

Consumers are increasingly drawn to products perceived as wholesome—those made with simple, natural ingredients and free from artificial chemicals. In response, both the meat industry and scientific communities are shifting toward minimizing synthetic substances by substituting them with naturally derived alternatives [8,12]. Consumer demand for natural food additives prompts scientists to research the development of alternative marinade ingredients [13,14]. They also take into account other criteria of consumers and producers, such as improving the tenderness or texture, taste, and shelf life of meat [5,15,16,17]. For this reason, various plant-based additives have become an increasingly attractive option compared to traditional preservatives and synthetic ingredients [8]. Traditional acidic agents like vinegar, lemon juice, and wine still dominate in marinades, but contemporary studies are turning attention to options such as fermented dairy and juices from fruits and vegetables [9]. Antioxidants, which naturally occur in fruits and vegetables, may reduce the risk of developing chronic human diseases, such as cardiovascular diseases, diabetes and cancer, and protect the health of consumers [18,19,20]. The subject of research by scientists was, among others, the effect of marinating with pineapple juice [18,21,22,23], ginger juice [24], kiwi [25,26], grape juice [15], mango juice [18], cherry and plum juice [9], tomato juice [13], cranberry juice [27] and the use of fermented milk derivatives [28] beer [29], and wine [30] for marinating meat. Vegetables such as onion, garlic [5,31], ginger [22], beetroot [32], pepper, etc. [17,33,34,35,36,37,38,39] as marinade additives to provide bioactive/health-promoting compounds such as beneficial phenols, flavonoids, polyphenols, etc. [31,32]. Plant-derived antioxidants play a key role in preserving meat by slowing down fat degradation processes, thereby prolonging its freshness [7,40].

Marinating is believed to boost product yield and enhance meat characteristics by making it more tender and juicy [41], preserving or intensifying color, enriching aroma and taste, minimizing undesirable flavors, as well as improving safety and prolonging shelf life through the inhibition of bacterial proliferation [9,42,43]. Marinades impart a crispy texture to muscle fibers, increase meat yield, improve water retention and textural properties, enhance meat flavor, and preserve food for a long time [44]. Many researchers have studied the effect of different marinades on the physicochemical and organoleptic properties of different types of meat, such as turkey and pheasant [42,45], chicken, pork [46], beef, and horse meat [18,47], goat meat [22], wild boar meat, roe deer [23].

A notable global market trend is the rising popularity of convenience foods, with marinated meat products ranking high among consumer favorites [8]. Incorporating natural ingredients in marinades not only shapes the product’s distinctive flavor and aroma but also promotes health benefits and enhances the dish’s authenticity [48,49]. Consumers are becoming more and more aware of the connection between the food they consume and their health. In addition, they are encouraged to avoid highly processed meat products, especially those heated to high temperatures during frying, baking or grilling [50,51]. Sous vide is considered to be a transformation of traditional cuisine into a more nutritious, healthier cuisine [52].

The growing demand for minimally processed food products, the properties of which are closer to fresh products, has led to the popularization of the sous vide technique in order to extend the shelf life and guarantee the quality of fresh food [53,54,55]. Sous vide cooking (vacuum cooking) is a new variant of the cooking technique usually used to produce high-quality food in the catering sector. Food is vacuum-packed in a thermostable plastic bag and then incubated in a water bath under controlled conditions of time and low temperatures (53–81 °C) [8]. This technique maintains the uniform quality of the meat and improves the organoleptic properties of the cooked meat. Meat cooked by the sous vide method is more tender and redder than meat cooked conventionally. The duration and temperature of cooking have a comparable effect on the physicochemical properties and palatability of the meat [56]. The mild heat treatment conditions used in sous vide cooking help to minimize the negative impact of heat treatment on nutrients such as proteins, lipids, and vitamins [57,58,59], and vacuum packaging prevents evaporative losses during cooking, preserves volatile compounds responsible for meat flavor, and minimizes the development of off-flavors [60,61]. This cooking method has been found to have the greatest advantages, such as the preservation of aromatic components, retention of favorable color, reduced thermal damage to proteins and lipids, loss of fluids, and essential nutrients such as vitamins and antioxidants [53,62]. These products stand out due to their enhanced nutritional content and superior sensory qualities, featuring bolder flavors and more complex aromas, which encourages some consumers to new and exotic culinary experiences [63]. In terms of food stability, sous vide cooking offers the advantage of extending the shelf life of products (the shelf life of sous vide-cooked products ranges from 6 to 42 days) by controlling microbial growth and the risk of microbial contamination compared to conventional cooking and chilling techniques [64]. Moreover, this method enables food service professionals to pre-cook meat dishes ahead of time, minimizing the chance of contamination during storage before they are served [65].

The application of low heat over prolonged periods contributes to tenderizing various meats, such as pork [66], beef [67], and lamb [68]. Notably, pork loin is especially prominent in Poland’s gastronomy. Traditional recipes commonly include pork loin dishes prepared through frying or baking. Heat treatment of meat causes protein denaturation, muscle fiber shrinkage, collagen solubilization and gelatinization, and considerable dehydration. It affects the mouthfeel of the meat. Most of the changes that occur in proteins during traditional heat treatment increase meat hardness [69]. As a result of moisture depletion, this type of meat becomes dry and experiences a reduction in essential nutrients [70]. This leads to a decline in consumer interest in these products. The low-temperature, vacuum-sealed cooking technique offers an alternative approach that can preserve or enhance the quality and market appeal of heat-processed pork [71,72]. The aim of this study was to evaluate the effect of marinade type and marinating time on the physicochemical and sensory properties of pork tenderloin cooked using the sous vide method.

## 2. Materials and Methods

The study material consisted of pork tenderloin purchased at a butcher’s shop. The purchased meat came from 15 half-carcasses 48 h after slaughter. The tenderloins were prepared for the study by removing the external fat tissue and divided into 9 groups; in each group, 15 slices were cut, each 2.5 cm thick. The experiment included 9 research variants (1 control group + 4 groups marinated for 2 h + 4 groups marinated for 12 h). The number of participants in each group was 15. Based on the pH and color measurements of the meat, the occurrence of PSE and DFD meat defects was excluded. The marinades were supplemented with 25 g of spices per 1 kg of meat. Liquid marinades were used in a weight ratio to meat of 1.5:1. Marinating using liquid marinades was carried out by immersion in tightly closed containers at a temperature of +3.5 °C.

The prepared meat slices were marinated in four different marinades. The following variants of marinades were used: (1) dry with a mixture of herbs, (2) wet based on kefir with a mixture of herbs, (3) wet based on dry red wine with a mixture of herbs, (4) wet based on 100% pineapple juice with a mixture of herbs. The herb mixture included spices such as iodized salt, black pepper, granulated garlic, ground red pepper, rosemary, juniper, and thyme. Four groups were marinated (one in each marinade) for 2 h, and four more (one in each marinade) were marinated for 12 h. The control sample was unmarinated meat. After a specified marinating time, the samples were transferred to thermostable bags and vacuum-packed. The prepared samples were cooked for 90 min in water set to 63 °C. After this process, the samples were cooled and subjected to laboratory tests.

### 2.1. Chemical Composition Analysis

The meat samples were comminuted using a Royal Catering RCMM-2000 grinder (Royal Catering, Butzbach, Germany), equipped with a 4 mm perforated plate to achieve the desired particle size. Then, the water content was assessed according to the guidelines specified in the PN-ISO 1442:2000 standard [73], the protein content according to the specifications specified in the PN-75/A-04018:2002 standard [74], and the fat content was analyzed according to the guidelines of the PN-ISO 1444:2000 standard [75]. Results are expressed in percentages.

### 2.2. Instrumental Evaluation of Meat Color

The CIE L*a*b* system was used to determine the color of pork tenderloin. For this purpose, the electronic colorimeter NR20XE EnviSense (EnviSense, Gdańśk, Poland) was used (light source D65, measuring head aperture 20 mm, calibration with white standard: L*—99.18, a*—0.07, b*—0.05). The determination was performed in triplicate. During the measurement, the colorimeter was coupled to a computer in which CQCS3 software ver. 3.4 EN (Shenzhen 3nh Technology Co., Ltd., Shenzhen, China) was installed.

### 2.3. Texture Profile Analysis (TPA)

Instrumental texture evaluation was performed on 2 cm cube-shaped meat samples. The texture parameters were tested according to the methodology described in the literature [76]. The following texture parameters were determined using the Texture Pro CT software (Shenzhen 3nh Technology Co., Ltd., Shenzhen, China): hardness cycle 1 [N], hardness cycle 2 [N], adhesiveness [mJ], elasticity [mm], chewiness [mJ], gumminess [N], resilience, and cohesiveness.

### 2.4. Sensory Evaluation of Meat

The assessment panel consisted of 10 non-smokers aged 40–55 years. The panel members were trained according to ISO 8586:2023 [77]. The assessment procedure was carried out according to ISO 13299:2016 [78]. The attributes assessed are presented in Table 1. The assessors were given coded samples, crackers and water to refresh their palates between the assessments of the samples. A five-point rating scale was used. Fleiss’ kappa coefficient of agreement was 0.31.

### 2.5. Statistical Analysis

Data normality was verified employing the Kolmogorov–Smirnov test, and variance equality was evaluated using the Brown–Forsythe test. The physicochemical characteristics and sensory assessment outcomes of the meat samples were subjected to multivariate analysis of variance (ANOVA) using the Statistica software (version 13.3; Stat Soft, Krakow, Poland). Marinating time and marinade type were treated as fixed effects, and feature parameters were treated as dependent variables. Mean values and standard errors of the means (SEM) are presented in the tables. To identify statistically significant differences between group means, Tukey’s HSD post hoc test was applied, considering results significant at a *p*-value below 0.05. 

## 3. Results and Discussion

In the pork tenderloin marinated using the dry method and in the kefir-based marinade for two hours, a slightly lower water content was found than in the control sample (Table 2). A higher water content was found in the pineapple juice and red wine marinade than in the control sample. This may confirm the findings of Ha et al. [79], who indicated that bromelain affects water retention in meat by hydrolyzing proteins, which may lead to increased water retention in meat.

Marinating for 12 h resulted in a reduction in the water content of the meat marinated in each marinade below the water content in the control group. The lower water content in the meat marinated in kefir can be explained by the fact that it leads to a small loss of water, which is consistent with the literature indicating the osmotic properties of fermented milk products [7]. The greatest difference was observed in the meat marinated in marinades based on pineapple juice and red wine. The fat content in meat marinated for 2 h in kefir, pineapple juice and red wine was similar, 1.99–2.02%, while the highest content was determined in the meat marinated dry (2.58%). Dry marinade can affect the fat content by absorbing moisture and fat from the meat surface. Latoch et al. [43] noticed that dry herbal marinades can affect the chemical composition of the meat, including the fat content.

Higher fat contents were found in meat marinated for 12 h, which was caused by a change in the proportions between the components due to a decrease in water content. Protein content ranged between 20.06% and 21.03%; longer marinating did not cause any significant changes in protein content. The lowest protein content was found in meat marinated in pineapple juice, which may confirm the reports of Ha et al. [79] on the activity of proteolytic enzymes contained in pineapple juice, which can break down proteins, which may lead to their loss, but at the same time may increase the availability of some amino acids.

The literature indicates that fermented milk products such as kefir may affect the fat content of meat by interacting with proteins and fats during the marinating process. For example, Latoch et al. [7] observed that marinating meat in kefir can lead to changes in fat content. Pineapple, on the other hand, contains the enzyme bromelain, which can break down proteins and fats, which may explain the initial increase and then decrease in fat content in meat. Ha et al. [79] indicated that bromelain has the ability to hydrolyze proteins and fats, which may affect their content in meat.

The texture properties depending on the type of marinade and marinating time are presented in Table 3. The values of the parameters hardness cycle 1 and hardness cycle 2 of meat cooked using the sous vide method after marinating for 2 h were significantly higher than in the control sample. The highest hardness of cycle 1 was characteristic of pork tenderloin marinated in kefir, while the pork tenderloin marinated using the dry method and in red wine had lower hardness. The literature indicates that dry marinades can lead to meat softening, which is consistent with the observations in our own studies [80]. Dry herbal marinade can affect the texture of meat through the osmotic effect of salt and the effect of phenolic compounds present in herbs, which can affect the structure of proteins. Pork tenderloin marinated in pineapple juice had the lowest hardness among the marinated samples. Similarly, Żochowska-Kujawska et al. [81] found an effect of marinating in wine, lemon juice, kefir or pineapple juice on reducing the hardness of game. These results are consistent with the findings of Ha et al. [79] on the use of bromelain in marinades, which confirm its effectiveness in reducing meat hardness. The enzyme bromelain contained in pineapple juice is characterized by strong proteolytic properties, breaking down muscle proteins and leading to meat tenderization. Other studies indicate a more significant hydrolytic effect on collagen than on myofibrillar proteins, which leads to better tenderization of tough meat [43,82,83].

Marinating for 12 h caused a significant decrease in hardness 1 and hardness 2, especially in the case of pork tenderloin marinated in kefir, a decrease in hardness 1 from 133.47 N after 2 h to 38.41 N after 12 h, hardness 2 decreased from 118.10 N to 33.70 N. The literature indicates that fermented milk products, such as kefir, contain lactic acid and proteolytic enzymes that can break down muscle proteins, leading to meat tenderization. Studies on marinating meat in fermented milk products confirm that longer marinating time results in greater meat tenderization [43]. A slightly smaller decrease was observed in the case of pork tenderloin marinated dry from 117.19 N to 49.74 N, and hardness 2 decreased from 100.40 N to 42.60 N after 12 h of marinating. A clear decrease was also observed for pork tenderloin marinated in pineapple juice. The hardness of pork tenderloin marinated in red wine was different, being lower after 2 h than in pork tenderloin marinated in kefir and dry marinated, but marinating for 12 h resulted in a slight decrease in meat hardness from 103.83 N to 100.89 N after 2 h and from 93.01 N to 91.39 N after 12 h. Latoch et al. [43] indicate that the effect of red wine, tannins and organic acids contained in it on meat texture through denaturation of surface proteins may be less pronounced compared to the enzymatic effect of marinades such as pineapple juice.

The adhesiveness of pork tenderloin after marinating for 2 h was significantly lower (from 0.27 mJ in dry-marinated meat to 1.57 mJ in kefir) compared to the control sample (4.15 mJ). Marinating for 12 h caused a significant increase in the adhesiveness of meat marinated in each of the marinades. Dry-marinated pork tenderloin was characterized by an adhesiveness of 1.0 mJ, and meat marinated in kefir had an adhesiveness of 4.19 mJ. The resilience of meat marinated for 2 h ranged from 0.12 to 0.16; after 12 h, the adhesiveness values ranged between 0.10 and 0.16 compared to the adhesiveness of the control sample at the level of 0.18. A similar level of the tested parameters was noted in the case of cohesion, which in the control sample was 0.41 and, in the meat, marinated for 2 h ranged from 0.37 to 0.44. The cohesion values after 12 h ranged from 0.31 to 0.38.

The elasticity of the control sample was 4.14 mm. The tenderloin marinated in kefir for 2 h had lower elasticity (3.98 mm). In the meat marinated using the dry method, the elasticity increased from 4.13 mm to 4.20 mm, while in the other marinades, a decrease in elasticity was noted.

The rubberiness of the pork tenderloin in the control sample was 26.35 N. Marinating for 2 h increased the value of this parameter from 40.04 N in the meat marinated in pineapple juice to 59.33 N in the pork tenderloin marinated in kefir but a longer marinating in kefir for 12 h caused a significant decrease in gumminess to 12.67 N. A similarly significant decrease in rubberiness from 51.17 N to 19.20 was observed in the pork tenderloin dry-marinated for 12 h. The marinating time in red wine caused a slight decrease in rubberiness.

The chewiness value in the control sample was 111.18 mJ. Marinating for 2 h caused a significant increase in this parameter. The lowest chewiness was characteristic of meat marinated in pineapple juice (178.04 mJ) and the highest in kefir (234.95 mJ). Marinating for 12 h caused a significant decrease in the chewiness of the pork tenderloin. The lowest chewiness was characteristic of pork tenderloin marinated for 12 h in kefir (47.51 mJ); pork tenderloin marinated in pineapple juice and dry marinated were characterized by higher similar values (91.08 mJ and 98.46 mJ, respectively). The highest chewiness after 12 h of marinating was characteristic of pork tenderloin marinated in red wine, and in this case, the decrease in chewiness compared to marinating for 2 h was the smallest in relation to the other marinades.

The value of the L* parameter in the control sample was 83.49 (Table 4). Marinating for 2 h caused a decrease in the mean values of this parameter. A greater decrease in lightness after 2 h was observed in pork tenderloin marinated in pineapple juice, red wine and dry marinating. Marinating for 12 h caused a significant increase in lightness (L*) in meat marinated in pineapple juice from 70.82 to 77.92 and a smaller increase in meat marinated in kefir. These results are consistent with the findings of Ha et al. [79], who indicate that short-term marinating may cause meat darkening, while longer may lead to lightening due to further protein degradation. Different conclusions were presented by Shah et al. [80], who indicate that proteolytic enzymes present in pineapple juice may cause the degradation of muscle proteins, which leads to changes in the structure and color of the meat, making it darker. Studies by Kargozari et al. [84] indicate that fermented milk products, such as kefir, may affect meat tenderness and juiciness, but their effect on color is less pronounced. According to the researchers, lactic acid may slightly denature surface proteins, which may lead to a slight lightening of the meat.

Marinating for 12 h in red wine caused a significant darkening of the meat (L* decreased from 71.10 to 64.65) and slightly less darkening of the dry-marinated meat.

The most important color parameter of meat and meat products is redness (a*), the most important factor determining consumer preferences [85,86]. The intensity of redness in cooked meat is inversely proportional to the degree of myoglobin denaturation [28,87]. Other studies have shown that sous-vide cooking can inhibit thermal denaturation of myoglobin due to the relatively lower heating temperature, which in turn can cause sous-vide-cooked meat to have an intense red color [64,88]. The control sample meat was characterized by the a* parameter of 8.69. Similar values were obtained for meat marinated for 2 h in kefir and dry-marinated (8.50 and 8.34). Meat marinated in pineapple juice was characterized by a higher a* value (9.78), while meat marinated in red wine had a lower a* than the control sample (7.67). After 12 h of marinating, the highest a* level was found in dry-marinated meat. This confirms the assumptions of Naveena and Mendiratta [89], who demonstrated the effect of herbs and spices and phenolic compounds contained in dry marinade on the stability of muscle pigments, such as myoglobin, leading to a more intense red color. Similarly, Shah et al. [80] indicate the antioxidant effect of phenolic compounds contained in herbs and spices, which protect myoglobin from oxidation and thus preserve the red color of the meat. Latoch et al. [28] confirm the beneficial effect of marinating time on the increase in redness. The longer the meat was marinated, the higher the a* values. The mechanism responsible for this indicates derivatives of milk protein hydrolysis bioactive peptides with antioxidant activity.

The intensity of redness is inversely proportional to the degree of myoglobin denaturation during marinating and processing. Karageorgou et al. [49] found that marinating pork and chicken in yogurt whey resulted in a decrease in redness (a*) in both raw and cooked meat. The b* value in the control sample was 9.44. In meat marinated for 2 h, the lowest b* value was found in meat marinated in red wine (8.94) and the highest in dry marinated meat (10.17). Longer marinating resulted in an increase in b* value except for meat marinated in red wine, which decreased from 8.94 to 7.80. Jayathilakan et al. [90] indicate that the cause of this condition is the anthocyanins contained in red wine, which can affect the color of the meat, giving it more yellowish shades in the initial stages of marinating. The studies by Suman and Poulson [91] suggest that wine polyphenols can bind to meat proteins, stabilizing its color and preventing myoglobin oxidation, which can lead to the preservation or even intensification of the red color of the meat. A different effect of acidic marinades on the color parameters of poultry meat was demonstrated by Çimen et al. [92]. They found that the highest L* value was observed in chicken breast marinated in lemon juice. Moreover, it was found that the b* value was the highest in the sample marinated in lemon juice. In another study, Unal et al. [93] found that marinating chicken breast meat in citric acid, lemon juice, and grapefruit juice significantly increased the L* and b* values. In the case of sous vide cooking, despite low temperatures, the use of acidic marinades can intensify the effects of denaturation compared to other marinades. As demonstrated by Horgan et al. [94] due to the influence of the acidic environment on lowering the thermal denaturation temperature of muscle proteins.

The results of the sensory evaluation of the marinated pork loin are presented in Table 5. The intensity of the smell of the control sample was assessed at 3.43 points, the meat marinated in kefir was assessed slightly lower (3.30 points), while the other meats marinated for 2 h received higher scores (3.63–4.10 points). The highest score of the meats marinated for 2 h was obtained by the dry-marinated meat. Marinating for 12 h resulted in more favorable scores for the discussed parameter in each case; however, similarly to the shorter marinating, the dry-marinating was assessed the best (4.87 points). The desirability of the smell showed similar relationships. All groups were characterized by higher scores compared to the control group. The scores after 2 h of marinating ranged from 3.53 points for the meat marinated in kefir to 4.17 points for the dry-marinated meat. After 12 h of marinating, the desirability of the smell of meat marinated in pineapple juice did not change (3.67 points); dry-marinated meat was rated at 4.93 points. Another analyzed feature was juiciness. In the control sample, juiciness was rated at 3.40 points. The juiciness of meat marinated for 2 h in kefir and pineapple juice was rated similarly (3.83 and 3.93 points), and after 12 h, the average scores were slightly lower (3.80 and 3.87 points). The juiciness of meat marinated in red wine and dry-marinated for 2 h was rated at 4.03 points, but after 12 h of marinating, the level of scores obtained increased significantly to 4.80 and 4.93 points. The tenderness of the meat was rated higher compared to juiciness. The control sample received a score of 3.73 points. The lowest scores among the meats marinated for 2 h were obtained by meat marinated in kefir (4.03 points) and dry marinated (4.07 points). The tenderness of meat marinated in pineapple juice and red wine was assessed at 4.30 points. Longer marinating caused a lower tenderness score in kefir and pineapple juice marinades, while marinating in red wine and dry marinated increased the tenderness scores to 4.77 points and 4.73 points, respectively.

The intensity of the palatability of the meat in the control sample was assessed at 3.67 points. Marinating for 2 h in all marinade variants was assessed at a similar level of 4.20 points. Longer marinating had a negative impact on the assessment of the palatability intensity of the meat marinated in kefir, a drop to (3.87 points), but contributed to a clear improvement in the assessment of the parameter in question in the case of marinating in red wine (4.80 points), dry (4.93 points). The desirability of the palatability of the control sample was assessed at 3.83 points. Among the different marinating variants, the lowest score was given to the meat marinated in pineapple juice (4.07 points) and the highest score to the meat marinated in red wine and dry (4.33 points). Marinating for 12 h caused a decrease in the assessment of the parameter in question in meat marinated in kefir (to 3.99 points) but a significant increase in the assessment in meat marinated in red wine and dry marinated (4.83 and 4.93 points). Many researchers have demonstrated a positive effect of marinades based on herbs and spices on the sensory quality of meat, as well as its taste, aroma and freshness [95,96,97,98,99]. Similarly, many authors report a beneficial effect of acidic marinades on the sensory quality of meat products [17,47,100,101], but in this case, it cannot be unequivocally confirmed. The reason may probably be a different method of heat treatment. The beneficial impact of marination on the quality of the meat was demonstrated by, among others, Sokołowicz and Augustyńska–Prejsnar [102], demonstrating better physicochemical properties and higher assessment of sensory properties, such as juiciness, tenderness and desirable smell. In other studies, Augustyńska–Prejsnar et al. [103] found that marinating with whey had a positive effect on the juiciness and tenderness of grilled meat.

## 4. Conclusions

The types of marinades used and the marinating time have an impact on some physicochemical and organoleptic properties of pork tenderloin. Changes in chemical composition during marination occur within a narrow range and are determined by the type of marinade used. More noticeable changes in chemical composition were found in the case of tenderloin marinated in pineapple juice. Marinating pork for 2 h increased texture parameters such as hardness, gumminess and chewiness compared to the control sample. In turn, extending the marinating time from 2 h to 12 h had an impact on reducing these texture parameters (except for the red wine marinade) and increasing adhesiveness. Marinating pork tenderloin darkens its color compared to the control sample, regardless of the type of marinade used. On the other hand, extending the marinating time from 2 h to 12 h causes a significant darkening of the meat, but only when marinated in wine and dry. Longer marinating time had a positive effect on all assessed sensory features of pork tenderloin marinated in wine and dry, while in the case of kefir marinade, such a situation occurred only for the intensity and desirability of smell. Moreover, pork tenderloin marinated in wine and dry obtained higher sensory evaluation scores for most of the analyzed features and extending the marinating time from 2 h to 12 h increased these scores even more.

## Figures and Tables

**Table 1:** The point scale for organoleptic evaluation of meat.

Specification	1	2	3	4	5
Tastiness—desirability	Undesirable	Hardly desirable	Neutral	Desirable	Highly desirable
Taste—intensity	Wery weakly perceptible	Weakly perceptible	Perceptible	Strong	Very strong
Tenderness	Very tough	Tough and fibrous	Slightly tender	Tender	Very tender
Juiciness]	Very dry	Dry	Slightly juicy	Juicy	Very juicy
Aroma—desirability	Undesirable	Hardly desirable	Neutral	Desirable	Highly desirable
Aroma—intensity	Very weakly perceptible	Weakly perceptible	Perceptible	Strong	Very strong

**Table 2:** The chemical composition of pork tenderloin is affected by different marinades and marination periods.

Parameters	Time [h]	Control Sample	Type of Marinade				SEM	ANOVA	*p*
			Kefir	Pineapple Juice	Red Wine	Dry Marinating			
Fat [%]	2 h	1.73 ^a^	1.99 ^b^	2.02 ^b^	2.01 ^b^	2.58 ^c^	0.03	M T T × M	*p* < 0.0001 0.05563 *p* < 0.0001
	12 h		2.62 ^c^	3.37 ^e^	2.11 ^b^	3.17 ^d^	0.02		
	SEM		0.02	0.01	0.02	0.04			
Water [%]	2 h	76.09 ^b^	75.85 ^b^	76.45 ^b^	75.74 ^b^	74.91 ^a^	0.10	M T T × M	*p* < 0.0001 0.07251 *p* < 0.0001
	12 h		75.33 ^a^	74.68 ^c^	75.79 ^b^	74.73 ^a^	0.02		
	SEM		0.02	0.01	0.02	0.19			
Protein [%]	2 h	20.91 ^a^	20.87 ^a^	20.06 ^b^	21.00 ^a^	20.46 ^ab^	0.10	M T T × M	0.2332 0.0411 *p* < 0.0001
	12 h		20.78 ^a^	20.60 ^ab^	20.87 ^a^	20.62 ^a^	0.01		
	SEM		0.02	0.01	0.01	0.19			

^a–e^—average values of the analyzed traits that are denoted by different letters within the same row show statistically significant differences (*p* ≤ 0.05); T—marinating time; M—type of marinade.

**Table 3:** Texture parameters depend on the type of marinade and marinating time.

Parameters	Time [h]	Control Sample	Type of Marinade				SEM	ANOVA	*p*
			Kefir	Pineapple Juice	Red Wine	Dry Marinating			
Hardness cycle 1 [N]	2 h	65.19 ^c^	133.47 ^a^	96.69 ^b^	103.82 ^b^	117.19 ^ab^	4.91	M T T × M	*p* < 0.0001 *p* < 0.0001 *p* < 0.0001
	12 h		38.41 ^c^	59.59 ^bc^	100.89 ^b^	49.74 ^c^	2.40		
	SEM		1.21	5.89	5.78	1.76			
Adhesiveness [mJ]	2 h	4.15 ^c^	1.57 ^a^	1.56 ^a^	1.06 ^a^	0.27 ^a^	0.47	M T T × M	0.0827 *p* < 0.0001 0.1070
	12 h		4.19 ^c^	3.09 ^c^	1.89 ^ab^	1.00 ^a^	0.47		
	SEM		0.48	0.30	0.49	0.13			
Resilience	2 h	0.18 ^a^	0.16 ^a^	0.12 ^b^	0.12 ^b^	0.13 ^a^	0.01	M T T × M	0.0624 0.1366 0.5121
	12 h		0.11 ^b^	0.13 ^b^	0.10 ^b^	0.16 ^a^	0.01		
	SEM		0.01	0.01	0.01	0.01			
Hardness cycle 2 [N]	2 h	54.92 ^c^	118.10 ^a^	83.33 ^b^	93.01 ^b^	100.40 ^ab^	4.68	M T T × M	*p* < 0.0001 0.0001 *p* < 0.0001
	12 h		33.70 ^c^	50.17 ^c^	91.39 ^b^	42.60 ^c^	2.01		
	SEM		1.37	5.32	5.32	1.56			
Cohesiveness	2 h	0.41 ^a^	0.44 ^a^	0.37 ^a^	0.41 ^a^	0.43 ^a^	0.01	M T T × M	0.0523 0.0547 0.0798
	12 h		0.34 ^b^	0.36 ^ab^	0.31 ^b^	0.38 ^ab^	0.01		
	SEM		0.01	0.02	0.01	0.01			
Springiness [mm]	2 h	4.14 ^a^	3.98 ^a^	4.19 ^a^	4.07 ^a^	4.13 ^a^	0.17	M T T × M	0.8678 0.0759 0.1261
	12 h		3.87 ^a^	3.85 ^a^	3.54 ^a^	4.20 ^a^	0.12		
	SEM		0.08	0.23	0.12	0.11			
Gumminess [N]	2 h	26.35 ^c^	59.33 ^a^	40.04 ^b^	44.72 ^b^	51.17 ^ab^	2.24	M T T × M	0.0002 *p* < 0.0001 *p* < 0.0001
	12 h		12.67 ^c^	22.33 ^c^	39.34 ^b^	19.20 ^c^	1.21		
	SEM		0.69	2.63	2.71	0.89			
Chewiness [mJ]	2 h	111.18 ± 7.03c	234.95 ^a^	178.04 ^b^	193.77 ^ab^	218.18 ^a^	10.07	M T T × M	0.0001 *p* < 0.0001 *p* < 0.0001
	12 h		47.51 ^d^	91.08 ^c^	166.23 ^b^	98.46 ^c^	5.77		
	SEM		3.24	12.35	11.57	4.53			

^a–d^—average values of the analyzed traits that are denoted by different letters within the same row show statistically significant differences (*p* ≤ 0.05); T—marinating time; M—type of marinade.

**Table 4:** Pork tenderloin color parameters depend on the type of marinade and marinating time.

Parameters	Time [h]	Control Sample	Type of Marinade				SEM	ANOVA	*p*
			Kefir	Pineapple Juice	Red Wine	Dry Marinating			
L*	2 h	83.49 ^ab^	78.45 ^a^	70.82 ^a^	71.10 ^a^	74.08 ^a^	2.65	M T T × M	0.0020 *p* < 0.0001 *p* < 0.0001
	12 h		79.96 ^a^	77.92 ^a^	64.65 ^b^	69.86 ^b^	2.84		
	SEM		1.22	4.25	3.71	1.80			
a*	2 h	8.69 ^ab^	8.50 ^a^	9.78 ^b^	7.67 ^a^	8.34 ^a^	0.31	M T T × M	*p* < 0.0001 *p* < 0.0001 *p* < 0.0001
	12 h		9.52 ^b^	8.06 ^a^	5.48 ^c^	11.22 ^b^	0.31		
	SEM		0.16	0.45	0.38	0.26			
b*	2 h	9.44 ^ab^	8.99 ^a^	9.18 ^b^	8.94 ^a^	10.17 ^ab^	0.33	M T T × M	*p* < 0.0001 *p* < 0.0001 0.0018
	12 h		10.76 ^d^	10.02 ^b^	7.80 ^c^	10.72 ^d^	0.34		
	SEM		0.14	0.46	0.49	0.25			

^a–d^—average values of the analyzed traits that are denoted by different letters within the same row show statistically significant differences (*p* ≤ 0.05); T—marinating time; M—type of marinade.

**Table 5:** Results of organoleptic evaluation of pork tenderloin depending on the type of marinade and marinating time [points].

Parameters	Time [h]	Control Sample	Type of Marinade				SEM	ANOVA	*p*
			Kefir	Pineapple Juice	Red Wine	Dry Marinating			
Aroma—intensity	2 h	3.43 ^ab^	3.30 ^a^	3.63 ^ab^	3.97 ^b^	4.10 ^b^	0.08	M T T × M	*p* < 0.0001 *p* < 0.0001 *p* < 0.0001
	12 h		3.93 ^b^	3.67 ^ab^	4.60 ^c^	4.87 ^c^	0.08		
	SEM		0.07	0.08	0.10	0.06			
Aroma—desirability	2 h	3.40 ^a^	3.53 ^a^	3.67 ^a^	3.97 ^b^	4.17 ^b^	0.09	M T T × M	*p* < 0.0001 *p* < 0.0001 *p* < 0.0001
	12 h		3.90 ^b^	3.67 ^ab^	4.80 ^c^	4.93 ^c^	0.05		
	SEM		0.07	0.08	0.08	0.06			
Juiciness	2 h	3.40 ^d^	3.83 ^a^	3.93 ^a^	4.03 ^a^	4.03 ^a^	0.09	M T T × M	*p* < 0.0001 *p* < 0.0001 *p* < 0.0001
	12 h		3.80 ^a^	3.87 ^a^	4.60 ^b^	4.93 ^c^	0.05		
	SEM		0.07	0.08	0.08	0.07			
Tenderness	2 h	3.73 ^a^	4.03 ^a^	4.30 ^a^	4.30 ^a^	4.07 ^a^	0.09	M T T × M	0.0512 *p* < 0.0001 *p* < 0.0001
	12 h		3.90 ^ab^	4.17 ^a^	4.77 ^c^	4.73 ^c^	0.06		
	SEM		0.06	0.08	0.09	0.08			
Taste—intensity	2 h	3.67 ^a^	4.20 ^b^	4.20 ^b^	4.20 ^b^	4.20 ^b^	0.09	MT T × M	0.1214 *p* < 0.0001 *p* < 0.0001
	12 h		3.87 ^a^	4.20 ^b^	4.80 ^c^	4.93 ^c^	0.07		
	SEM		0.07	0.10	0.09	0.06			
Tastiness—desirability	2	3.83 ^a^	4.20 ^b^	4.07 ^b^	4.33 ^ab^	4.33 ^ab^	0.08	M T T × M	0.0735 *p* < 0.0001 *p* < 0.0001
	12		3.99 ^b^	4.13 ^b^	4.83 ^c^	4.93 ^c^	0.07		
	SEM		0.07	0.09	0.08	0.06			

^a–d^—average values of the analyzed traits that are denoted by different letters within the same row show statistically significant differences (*p* ≤ 0.05); T—marinating time; M—type of marinade.

## Data Availability

The original contributions presented in the study are included in the article; further inquiries can be directed to the corresponding authors.
